# Nano SiO_2_ and MgO Improve the Properties of Porous β-TCP Scaffolds via Advanced Manufacturing Technology

**DOI:** 10.3390/ijms16046818

**Published:** 2015-03-25

**Authors:** Chengde Gao, Pingpin Wei, Pei Feng, Tao Xiao, Cijun Shuai, Shuping Peng

**Affiliations:** 1 Hunan Provincial Tumor Hospital and the Affiliated Tumor Hospital of Xiangya School of Medicine, Central South University, Changsha 410013, China; E-Mail: gaochengde@csu.edu.cn; 2 State Key Laboratory of High Performance Complex Manufacturing, Central South University, Changsha 410083, China; E-Mail: fengpei@csu.edu.cn; 3 Cancer Research Institute, Central South University, Changsha 410078, China; E-Mail: aishangnidelian@sina.com; 4 Orthopedic Biomedical Materials Institute, Central South University, Changsha 410083, China; E-Mail: xiaotaoxyl@163.com; 5 Department of Orthopedics, the Second Xiangya Hospital, Central South University, Changsha 410011, China

**Keywords:** β-tricalcium phosphate, SiO_2_, MgO, selective laser sintering, scaffolds

## Abstract

Nano SiO_2_ and MgO particles were incorporated into β-tricalcium phosphate (β-TCP) scaffolds to improve the mechanical and biological properties. The porous cylindrical β-TCP scaffolds doped with 0.5 wt % SiO_2_, 1.0 wt % MgO, 0.5 wt % SiO_2_ + 1.0 wt % MgO were fabricated via selective laser sintering respectively and undoped β-TCP scaffold was also prepared as control. The phase composition and mechanical strength of the scaffolds were evaluated. X-ray diffraction analysis indicated that the phase transformation from β-TCP to α-TCP was inhibited after the addition of MgO. The compressive strength of scaffold was improved from 3.12 ± 0.36 MPa (β-TCP) to 5.74 ± 0.62 MPa (β-TCP/SiO_2_), 9.02 ± 0.55 MPa (β-TCP/MgO) and 10.43 ± 0.28 MPa (β-TCP/SiO_2_/MgO), respectively. The weight loss and apatite-forming ability of the scaffolds were evaluated by soaking them in simulated body fluid. The results demonstrated that both SiO_2_ and MgO dopings slowed down the degradation rate and improved the bioactivity of β-TCP scaffolds. *In vitro* cell culture studies indicated that SiO_2_ and MgO dopings facilitated cell attachment and proliferation. Combined addition of SiO_2_ and MgO were found optimal in enhancing both the mechanical and biological properties of β-TCP scaffold.

## 1. Introduction

β-Tricalcium phosphate (β-TCP, β-Ca_3_(PO_4_)_2_) has been widely used as bone substitute material for bone regeneration because of its excellent biocompatibility and bioactivity [1]. However, the poor mechanical strength and low resistance to crack-growth propagation hinder its applications in load-bearing applications [2]. Besides, the too fast and uncontrollable degradation rate is another major shortcoming of β-TCP, which makes it difficult to keep mechanical integrities before the bone defects are sufficiently healed [3]. Thus, the burning question is to improve the mechanical strength and decrease the degradation rate of β-TCP simultaneously.

Various approaches have been explored to improve the properties of bioceramic, such as incorporation of sintering additives [4], polarization [5] and surface coating [6,7]. Introducing trace minerals into bioceramic has drawn scientific interest since it is effective in improving the mechanical properties and biological responses [8]. Researchers have developed β-TCP composites doped with CaO, Ag_2_O, TiO_2_, MgO, SrO, SiO_2_ and ZnO minerals for desired mechanical properties [9,10]. Silicon (Si) is an important trace element for bone tissue formation [11]. It can stimulate cellular activities such as the proliferation and differentiation of osteoblast-like cells as well as mineralization [12]. Magnesium (Mg) is also one of the most essential elements in nature bone [13]. It can affect mineral metabolism and stimulate new bone formation [14].

Carlisle verified that Si (up to 0.5 wt %) often localized in active growth areas and played an important role in bone formation and calcification [15]. SiO_2_/ZnO was doped into β-TCP scaffolds by Fielding *et al.* (2012). They proved that SiO_2_ and ZnO dopings increased the mechanical strength and facilitated cellular proliferation [16]. Bandyopadhyay *et al.* studied the effect of ZnO, SiO_2_ and SrO dopings on the mechanical and biological properties of β-TCP ceramics. And it was concluded that the dopings could tailor the strength and degradation behavior [17]. Ryu *et al.* introduced MgO into dense hydroxyapatite (HA)/β-TCP ceramics to improve the sintering ability. The results showed a transgranular fracture mode and enhanced mechanical properties of HA/β-TCP ceramics doped with 1.0 wt % MgO [18].

Besides, an interconnected porous structure in bone scaffold is essential to facilitate cell infiltration, bone growth and vascularization [19]. So far, various techniques have been developed to fabricate porous scaffolds, including solvent casting, melt molding and gas foaming. Nevertheless, it is difficult to achieve accurate anatomical geometries and controlled spatial structures by these methods. As an advanced manufacturing technology, selective laser sintering (SLS) can fabricate customized implants with complex geometry and internal porous structure according to the bone defect sites [20]. These characteristics make SLS a promising technique in the development of porous bone scaffolds [21].

In light of the present scenario, small quantities of nano SiO_2_ (0.5 wt %) and nano MgO (1.0 wt %) were used as dopants to improve the mechanical and biological properties of β-TCP scaffolds. The scaffolds were fabricated by SLS and characterized in terms of phase composition, morphology and compressive strength. The biodegradation behavior was evaluated by immersing the scaffolds in simulated body fluid (SBF) for 2 and 4 weeks. In addition, the human osteosarcoma MG-63 cells were cultured on the scaffolds to assess cell proliferation *in vitro*.

## 2. Results and Discussion

### 2.1. Characterization of the Scaffolds

**Figure 1:**
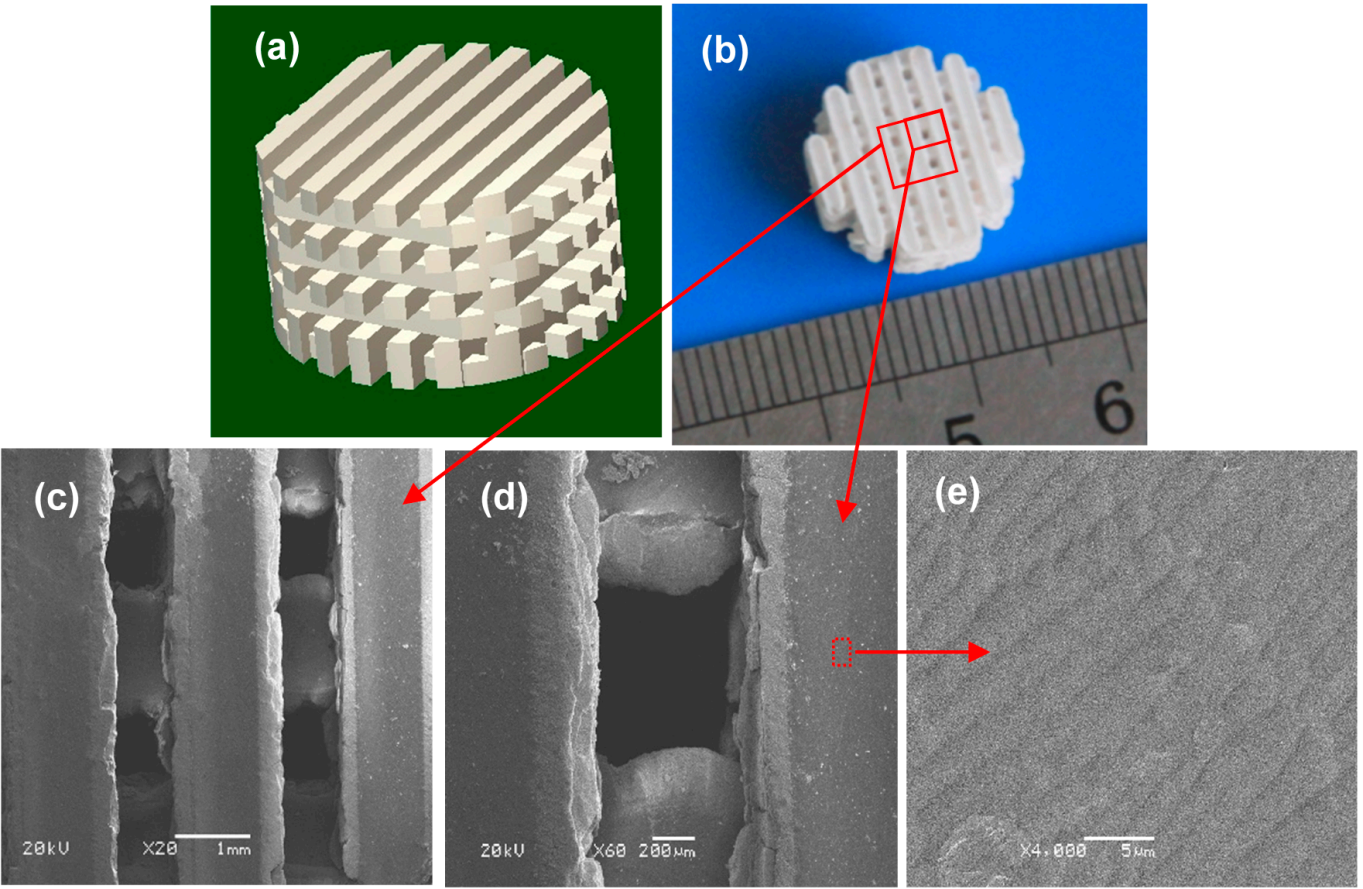
(**a**) Computer-aided design (CAD) model of the scaffold; (**b**) Porous scaffold (machined into a cylinder); (**c**–**e**) Scanning electron microscopy (SEM) micrographs of the interconnected network channels.

Cylindrical scaffold model with a 3D orthogonal porous architecture was designed using SolidWorks, as shown in Figure 1a. The model was designed with radius 7 mm, height 8 mm and interconnected square channels size 0.6 × 0.6 mm^2^. The designed porosity for all scaffolds was 48.3%. A representative cylindrical scaffold was fabricated via SLS (Figure 1b) with laser power 12 W, scan speed 100 mm/min, layer thickness 0.1 mm and laser beam spot size 800 μm. The scaffold had an intact structure and possessed good handling stability. The internal channel was observed by SEM, as shown in Figure 1c,d. The scaffold possessed interconnected porosity which was ~600 μm in *X*-*Y* plane. Besides, good connections between pore walls and uniform network structure were observed. Surface morphologies of all the scaffolds revealed a highly dense structure (Figure 1e).

### 2.2. Phase Identification

**Figure 2:**
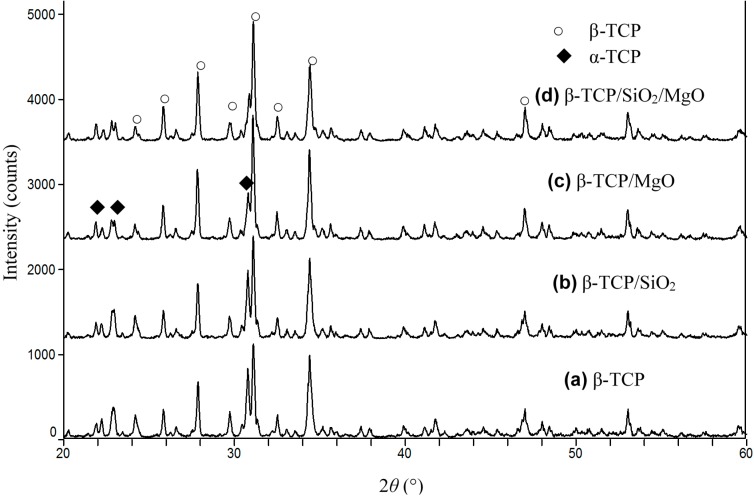
X-ray diffraction (XRD) patterns of (**a**) β-tricalcium phosphate (β-TCP); (**b**) β-TCP/SiO_2_; (**c**) β-TCP/MgO; and (**d**) β-TCP/SiO_2_/MgO.

X-ray diffraction (XRD) patterns from sintered scaffolds were presented in Figure 2. It can be seen that the scaffolds were composed of β-TCP and α-TCP after sintering. There were no peaks for SiO_2_ and MgO due to the low weight percent in β-TCP ceramic matrix. α-TCP formed in all sintered scaffolds since β-TCP started transforming to α-TCP above the temperature of 1180 °C, as demonstrated by Miao *et al.* [22]. A quantitative comparison of the integrated intensities of the peaks for β-TCP after sintering was given in Table 1. The major phase of scaffolds remained unchanged as β-TCP even after the addition of SiO_2_ or MgO. The doping of 1 wt % MgO decreased the amount of α-TCP in the scaffolds because MgO increased the β-α transformation temperature, which was in line with the study by Bose *et al.* [23]. However, the scaffold doped with 0.5 wt % SiO_2_ did not show noticeable difference in the amount of β-TCP as compared with β-TCP scaffold.

The apparent densities of the scaffolds were determined in water using Archimedes principle and then normalized with the theoretical density of β-TCP (3.07 g/cm^3^) to obtain the relative density of sintered scaffolds (Table 1). β-TCP scaffold had a relative density of 90.21% ± 1.53%, whereas the doping of SiO_2_ increased the density slightly (91.35% ± 1.46%). The relative density of β-TCP/MgO and β-TCP/SiO_2_/MgO increased to 93.28% ± 1.08% and 95.53% ± 0.94%, respectively. The phase transformation from β-TCP to α-TCP prevented further densification due to the volume expansion. It was concluded that MgO could inhibit the phase transformation and improve the densification of β-TCP scaffolds.

**Table 1:** Weight percents of β-tricalcium phosphate (β-TCP) after sintering and relative densities of scaffolds.

Compositions	β-TCP	β-TCP/SiO_2_	β-TCP/MgO	β-TCP/SiO_2_/MgO
Weight percent of β-TCP (%)	85.5	87.0	93.6	93.3
Relative density (%)	90.21 ± 1.53	91.35 ± 1.46	93.28 ± 1.08	95.53 ± 0.94

### 2.3. Mechanical Properties

**Figure 3:**
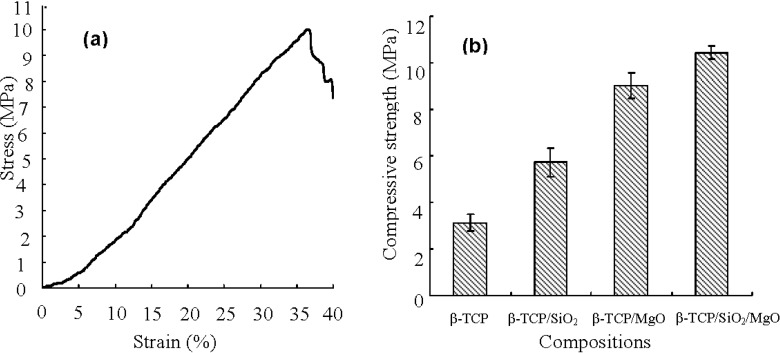
(**a**) A typical stress-strain plot of the scaffolds; and (**b**) Compressive strength of the scaffolds.

The typical stress-strain curve from compression testing was shown in Figure 3a. The trends of stress-strain curves for all scaffolds were in agreement with the behavior of ceramics. The compressive strength of the scaffolds was shown in Figure 3b. The β-TCP scaffold had a compressive strength of 3.12 ± 0.36 MPa. It was observed that both SiO_2_ and MgO dopings increased the compressive strength of β-TCP scaffold. The compressive strength of β-TCP/SiO_2_ scaffold increased to 5.74 ± 0.62 MPa. The compressive strength for β-TCP/MgO scaffold was higher than that of β-TCP/SiO_2_ scaffold, attaining 9.02 ± 0.55 MPa. The β-TCP/SiO_2_/MgO scaffolds exhibited the highest compressive strength of 10.43 ± 0.28 MPa, which was comparable to that of cancellous bone (0.1–16 MPa) [24]. The results clearly indicated that 1.0 wt % MgO was more beneficial in improving compressive strength of β-TCP scaffold than 0.5 wt % SiO_2_. Higher compressive strength for β-TCP/MgO and β-TCP/SiO_2_/MgO scaffolds might be ascribed to the reduced formation of α-TCP phase and increased density after MgO doping, thereby significantly improving the mechanical properties of the scaffold. Impens *et al.* prepared β-TCP scaffolds utilizing gel-casting method. The scaffolds had a porosity of 80% and exhibited a compressive strength of 2.5 MPa [25], which was lower than that of the scaffolds fabricated in this study.

### 2.4. Mineralization in Simulated Body Fluid (SBF)

**Figure 4:**
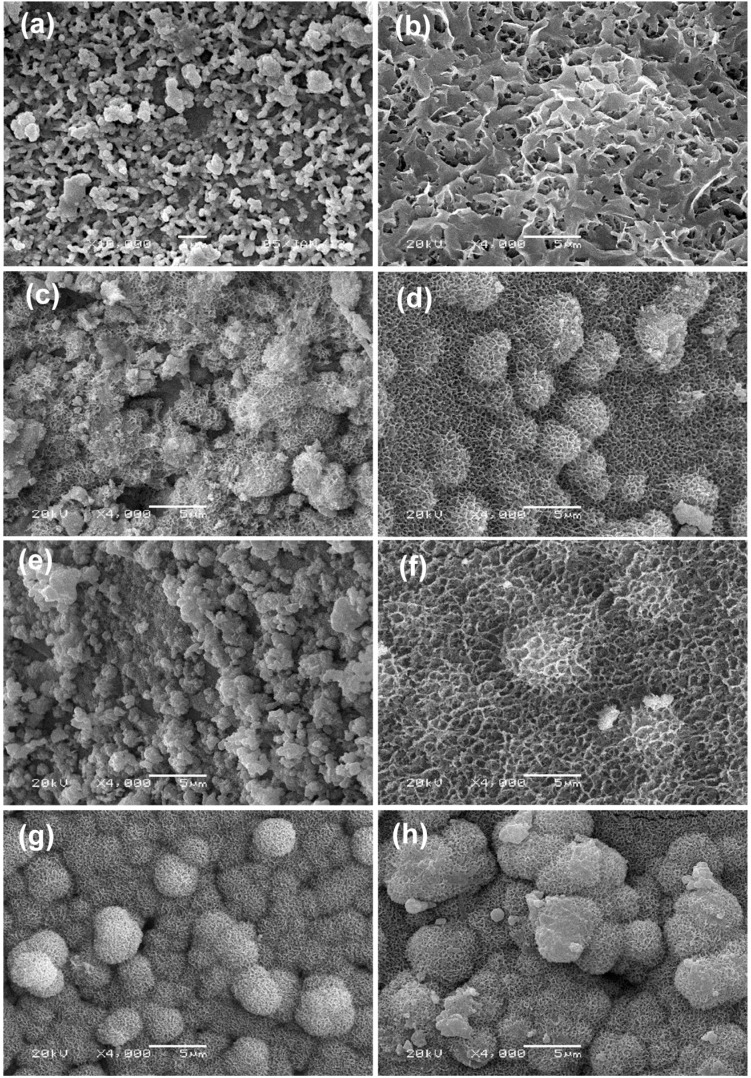
SEM images of the scaffolds after immersion in simulated body fluid (SBF) for 2 weeks and 4 weeks: (**a**,**b**) β-TCP; (**c**,**d**) β-TCP/SiO_2_; (**e**,**f**) β-TCP/MgO and (**g**,**h**) β-TCP/SiO_2_/MgO. 2 weeks: (**a**,**c**,**e**,**g**); 4 weeks: (**b**,**d**,**f**,**h**).

The bone-like layer on scaffold surfaces was revealed in Figure 4. All the scaffolds presented smooth and dense surfaces before immersion in SBF. Bone-like apatite was observed on all the scaffold surfaces with different morphology. There were some apatite granules on the β-TCP scaffold after soaking in SBF for 2 weeks, as shown in Figure 4a. The apatite layer was thin and the original contour of the specimen was still visible. The apatite nucleated from these individual granules and formed a compact layer with time. After soaking in SBF for 4 weeks, the surface was completely covered with a plate-like layer (Figure 4b).

The surface of β-TCP/SiO_2_ scaffold was covered with petal-like apatite layer after soaked for 2 weeks (Figure 4c). For 4 weeks, some spherical particles with sizes ranging from 2 to 3 μm nucleated on the petal-like apatite layer (Figure 4d). Moreover, these particles were also made up of many fine petal-like crystallites. Spherical petal-like particles on the plate-like apatite layer indicated a fast bone-forming speed on this scaffold. These results demonstrated that SiO_2_ enhanced the bone-like apatite forming ability of β-TCP scaffolds. The surface of β-TCP/MgO scaffold showed complete coverage by aggregated granules of apatite after 2 weeks in SBF. MgO doping accelerated the apatite layer formation when compared with pure β-TCP. After 4 weeks, the surface of β-TCP/MgO scaffold also formed plate-like layer and some spherical petal-like particles started to nucleate on the petal-like apatite layer (Figure 4f). The apatite morphology on β-TCP/SiO_2_/MgO scaffold after 2 weeks was similar to that on β-TCP/SiO_2_ scaffold after 4 weeks. The main difference was due to the fact that much more spherical crystals attached on the β-TCP/SiO_2_/MgO surface than on β-TCP/SiO_2_ for 2 weeks in SBF (Figure 4g). The spherical crystals continued to grow in solution and the size increased to 5 μm after 4 weeks (Figure 4h). Thus both SiO_2_ and MgO could accelerate the formation of bone-like apatite on β-TCP scaffold and β-TCP/SiO_2_/MgO scaffold exhibited the fastest mineral forming ability.

**Figure 5:**
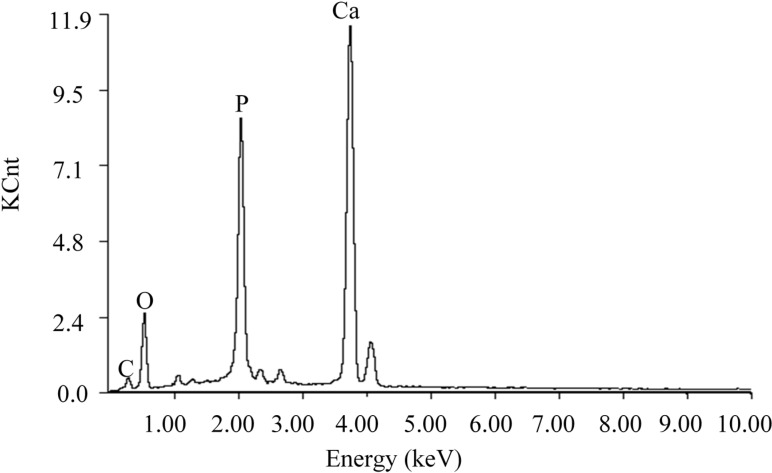
Energy dispersive spectrometer (EDS) analysis of the mineral layers on the scaffold surface after immersed in SBF.

The mineral layers on the scaffold surface were confirmed by EDS analysis (Figure 5). This demonstrated that the mineral layer was mainly composed of calcium, phosphorus and oxygen with a Ca/P mole ratio of 1.675. The Ca/P mole ratio of mineral component was similar to hydroxy-carbonate apatite (HCA) (Ca/P mole ratio: 1.67).

### 2.5. Weight Loss

**Figure 6:**
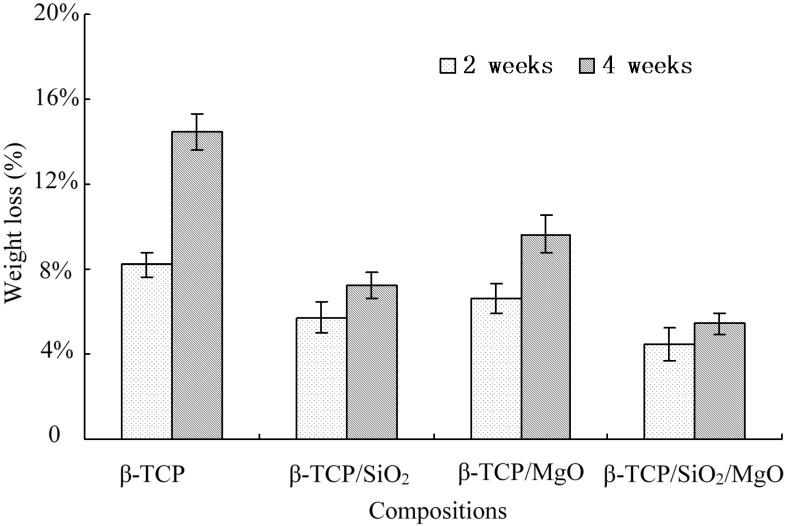
Weight loss (in wt %) of the scaffolds in SBF.

The scaffolds underwent both apatite layer formation and degradation processes in SBF. The weight change percent of scaffolds in SBF after 2 and 4 weeks were shown in Figure 6. The β-TCP scaffold had the highest degradation rate with weight loss of 8.19 ± 0.56 wt % after 2 weeks and 14.46 ± 0.82 wt % after 4 weeks. Compared with β-TCP scaffold, the composite scaffolds (β-TCP/SiO_2_, β-TCP/MgO and β-TCP/SiO_2_/MgO) displayed lower degradation rates. The weight losses were 5.70 ± 0.72 wt %, 6.62 ± 0.68 wt %, and 4.43 ± 0.76 wt % respectively after 2 weeks. And β-TCP/SiO_2_/MgO showed the lowest weight loss of 5.44 ± 0.52 wt % (4 weeks). The weight loss results demonstrated that the degradation dominated over apatite formation for all scaffolds. Nevertheless, the addition of SiO_2_ and MgO slowed down the degradation of β-TCP scaffolds. This was because Si and Mg increased the mineralization rate in SBF. Si and Mg acted as nucleation or created a favorable condition for apatite formation and at the same time slowed down the dissolution process *in vitro* [26]. Weight loss of β-TCP/SiO_2_/MgO scaffold was significantly lower than that of β-TCP scaffold. As Ca ions were released to the solution, hydrogen ions formed a hydrated silica leaching layer containing Si–OH groups on the scaffold surface, which acted as a catalyzing agent for the nucleation and growth of bone-like apatite layer. Li *et al.* found that SiO_2_ can form a Si–OH functional layer easily *in vitro* and thereby enhance the mineralization of apatite-like phase. It was believed that the fast formation of bone-like layer on the surfaces of these scaffolds was closely related to the increasingly provided silanol groups [27].

The high degradation rate of β-TCP made it difficult to match the growth rate of new bone tissues. Results of this study revealed that SiO_2_ and MgO could be used as additives to adjust the biodegradation characteristics of β-TCP scaffold.

### 2.6. Cell Attachment and Proliferation

**Figure 7:**
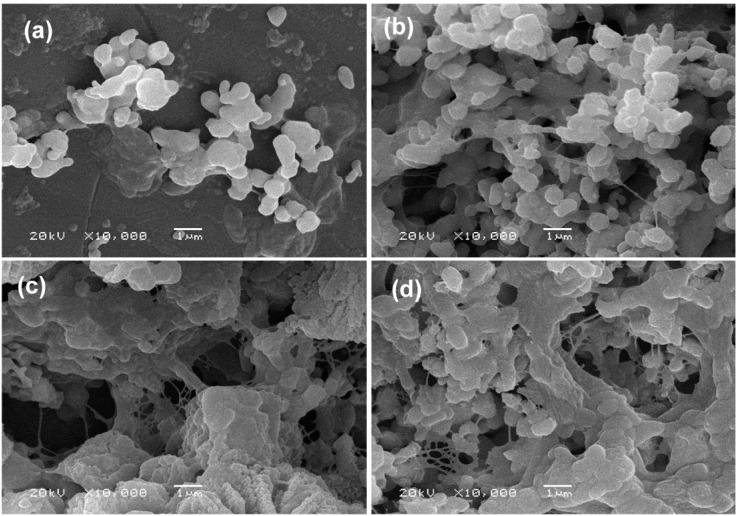
SEM morphology of MG-63 cells on the scaffolds for 7 days. (**a**) β-TCP; (**b**) β-TCP/SiO_2_; (**c**) β-TCP/MgO; and (**d**) β-TCP/SiO_2_/MgO.

For *in vitro* biological evaluation, MG-63 cells were cultured and seeded on each scaffold. Morphologies of MG-63 cells on the scaffolds after 7 days were shown in Figure 7. A few cells were distributed on the β-TCP scaffold and exhibited elongated, flattened morphology with few filopodia extensions (Figure 7a). More cells attached on the surface of β-TCP/SiO_2_ compared with β-TCP (Figure 7b). Cells produced much filopodia and extracellular matrix (ECM) extensions. The results demonstrated that SiO_2_ enhanced the cell adhesion and differentiation. Cell proliferation on β-TCP/MgO scaffold surface was significantly increased, as shown in Figure 7c. The amount of visible cells on SiO_2_/MgO doped scaffold was significantly greater than that on other scaffolds. A large number of cells attached on the β-TCP/SiO_2_/MgO scaffold with numerous lamellipodia and filopodia extensions, indicating excellent cell attachment and proliferation (Figure 7d).

The research findings suggested good cell coverage on the surfaces of doped scaffolds. Combined addition of SiO_2_ and MgO into β-TCP scaffolds improved cell attachment and proliferation, which was favorable for bone regeneration. Furthermore, the incorporation of SiO_2_ and MgO was beneficial to achieve combined benefits of matching bone chemistry.

## 3. Experimental Section

### 3.1. Materials

β-TCP powder with an average particle size of 0.2 μm was obtained from Kunshan Chinese Technology New Materials Co. (Kunshan, China). Nano SiO_2_ (99% purity, particle size 10–30 nm) and nano MgO (99.9% purity, average particle size 20 nm) were purchased from Nanjing Emperor Nano Material Co. (Nanjing, China). Pure β-TCP powder (β-TCP), β-TCP powder doped with 0.5 wt % SiO_2_ (β-TCP/SiO_2_), β-TCP powder doped with 1.0 wt % MgO (β-TCP/MgO), β-TCP powder doped with 0.5 wt % SiO_2_ and 1.0 wt % MgO (β-TCP/SiO_2_/MgO) were prepared by mechanical mixing, respectively. The amounts were chosen according to previous research.

### 3.2. Scaffold Fabrication and Characterization

The powders of different compositions (β-TCP, β-TCP/SiO_2_, β-TCP/MgO and β-TCP/SiO_2_/MgO) were used to fabricate scaffolds. All scaffolds were fabricated on a home-developed SLS system using a 100 W CO_2_ laser (model: Firestar^®^ t-Series, Synrad Co., Mukilteo, WA, USA) with wavelength of 10.6 μm [28]. In the SLS process, the powder was deposited onto the elevating platform to form a thin powder layer. Then the powder layer was selectively sintered and bonded to form a solid layer. After one layer was finished, the elevating platform was moved down by the thickness of a layer. Then successive layers were processed in this manner until the scaffold was completed. After the SLS process, the scaffolds were cooled to room temperature. Excess powders were brushed off and the scaffolds were cleaned using compressed air. The laser sintering parameters were kept constant during the sintering process: Laser power 12 W, scan speed 100 mm/min, layer thickness 0.1 mm and laser beam spot size 800 μm.

The scaffolds were coated with gold for SEM analysis (JSM-6490LV, JEOL Co., Tokyo, Japan) to investigate the surface morphologies of the scaffolds. The compressive strengths of the scaffolds were determined on a mechanical tester (WD-D1, Shanghai Zhuoji Instruments Co., Shanghai, China) with a constant cross-head speed of 0.4 mm/min. Compressive strength was calculated from the stress-strain curve based on the maximum load recorded and scaffold dimension. For each group, five scaffolds with radius 7 mm and height 8 mm were tested to ensure reproducibility. The data were analyzed statistically.

### 3.3. X-ray Diffraction (XRD)

The phases of the scaffolds were characterized with XRD diffractometer (model D8-ADVANCE, Bruker AXS Inc., Karlsruhe, Germany) using Cu-Kα radiation at 40 mA and 40 kV. Samples were grinded into powders and tested with 2θ values from 20° to 60° at a step size of 0.02°. Phases were identified by comparing the diffraction patterns of samples with the standard PDF cards: α-TCP (JCPDS No. 9-348), β-TCP (JCPDS No. 9-169), MgO (JCPDS No. 45-946), SiO_2_ (JCPDS No. 76-1390). The relative amounts of β-TCP after sintering were determined by integrated intensities of their peaks in XRD patterns. Reference intensity ratio (RIR) method was employed to identify and quantify the weight percent of β-TCP in the sintered samples according to Equation (1):

(1) $$X_{i}=\;\frac{I_{i}}{RIR_{i}\sum_{j=1}^{n}\frac{I_{j}}{RIR_{j}}}\;\times 100\%$$

where *X_i_* is the weight percent of crystalline phase *i*, *RIR_i_* and *RIR_j_* are the reference intensity ratios of crystalline phase *i* and *j*, *I_i_* and *I_j_* are the integrated intensities of the diffraction peaks of phase *i* and *j*, and *n* is the number of crystalline phases.

### 3.4. The Degradability and Bioactivity

All scaffolds were immersed in SBF solution to investigate their degradation behavior and bioactivity ability. SBF solution with ionic composition (in units of mM, 142 Na^+^, 5.0 K^+^, 1.5 Mg^2+^, 2.5 Ca^2+^, 148 Cl^−^, 4.2 HCO_3_^−^, 1.0 HPO_4_^2−^ and 0.5 SO_4_^2−^) was almost equal to that of human plasma and buffered at similar pH. The scaffolds were immersed into the SBF solution at 37 °C for 2 and 4 weeks. The solution was changed every 3 days during the immersion process. At each time point, four samples of each composition were removed, washed with distilled water, and then dried at 65 °C in an oven for 48 h. After the drying process, the weight of each sample was carefully recorded and compared with their net weight before immersion. The morphology of apatite layers on scaffold surfaces was observed under SEM. The mineral layer was also analyzed by energy dispersive spectrometer (EDS) (Neptune XM4, EDAX Inc., Mahwah, NJ, USA).

### 3.5. In Vitro Cell Culture

Human osteosarcoma MG-63 cells were used to explore the cell-materials interactions. Before seeding, the scaffolds were sterilized with 70% ethanol for 30 min and then further sterilized under UV light for 30 min, followed by drying under sterile conditions at room temperature for 2 h. Then the scaffolds were washed and immersed in phosphate-buffered saline (PBS) for 24 h. Cells were cultured in α-MEM medium (Hyclone, Logan, UT, USA) supplemented with 10% FBS (Gibco, Carlsbad, CA, USA) and 100 U/mL penicillin/streptomycin (Hyclone) at 37 °C under 5% CO_2_ condition. Digested cells with trypsine (Biological Industries, Kibbutz Beit Haemek, Israel) were then directly seeded on the scaffolds after removing PBS solution completely. To avoid cell leakage during cell seeding, the concentrated MG-63 suspension was dropped onto the scaffolds and cultured for 1 h to allow the attachment of cells onto the surfaces. Then the medium was supplemented to 1 mL well. The medium was changed every 3 days. The medium was removed after 7 days, and the scaffolds with attached cells were washed with PBS and dehydrated in graded ethanol (30%–100%) and Freon. The fully dried samples were coated with a thin gold film and the morphology of the cells was analyzed with SEM (JSM-6490LV, JEOL Co., Tokyo, Japan). For each group, five scaffolds with radius 7 mm and height 8 mm were cell-cultured and tested to ensure reproducibility.

## 4. Conclusions

Nano SiO_2_ and MgO were used to improve the mechanical and biological properties of β-TCP scaffolds. Porous cylindrical scaffolds were successfully fabricated via SLS technique. MgO improved the thermal stability and reduced the phase transformation from β to α-TCP. The compressive strength of β-TCP scaffold doped with SiO_2_/MgO showed the maximum increase from 3.12 ± 0.36 to 10.43 ± 0.28 MPa. *In vitro* mineralization study in SBF indicated that SiO_2_ and MgO could decrease the biodegradation rate of β-TCP scaffold and facilitate apatite growth. *In vitro* cell culture study demonstrated that SiO_2_ and MgO dopings promoted the cell attachment and proliferation of β-TCP scaffold. Thus, β-TCP scaffold doped with SiO_2_ and MgO was a potential candidate for bone substitutes applied in bone tissue engineering.

## References

[1] Cao H., Kuboyama N. (2010). A biodegradable porous composite scaffold of PGA/β-TCP for bone tissue engineering. Bone.

[2] Tricoteaux A., Rguiti E., Chicot D., Boilet L., Descamps M., Leriche A., Lesage J. (2011). Influence of porosity on the mechanical properties of microporous β-TCP bioceramics by usual and instrumented Vickers microindentation. J. Eur. Ceram. Soc..

[3] Wang C., Xue Y., Lin K., Lu J., Chang J., Sun J. (2012). The enhancement of bone regeneration by a combination of osteoconductivity and osteostimulation using β-CaSiO_3_/β-Ca_3_(PO_4_)_2_ composite bioceramics. Acta Biomater..

[4] Bhatt H.A., Kalita S.J. (2007). Influence of oxide-based sintering additives on densification and mechanical behavior of tricalcium phosphate (TCP). J. Mater. Sci. Mater. Med..

[5] Tarafder S., Bodhak S., Bandyopadhyay A., Bose S. (2011). Effect of electrical polarization and composition of biphasic calcium phosphates on early stage osteoblast interactions. J. Biomed. Mater. Res. Part B Appl. Biomater..

[6] Obata A., Kasuga T. (2009). SiO_2_-CaO-P_2_O_5_ sol-gel-derived glass coating on porous β-tricalcium phosphate ceramics. J. Ceram. Soc. Jpn..

[7] Yu X., Wei M. (2013). Cellular performance comparison of biomimetic calcium phosphate coating and alkaline-treated titanium surface. BioMed Res. Int..

[8] Zhang M., Wu C., Lin K., Fan W., Chen L., Xiao Y., Chang J. (2012). Biological responses of human bone marrow mesenchymal stem cells to Sr-M-Si (M = Zn, Mg) silicate bioceramics. J. Biomed. Mater. Res. Part A.

[9] Tarafder S., Davies N.M., Bandyopadhyay A., Bose S. (2013). 3D printed tricalcium phosphate bone tissue engineering scaffolds: Effect of SrO and MgO doping on in vivo osteogenesis in a rat distal femoral defect model. Biomater. Sci..

[10] Hu H., Liu X., Ding C. (2010). Preparation and *in vitro* evaluation of nanostructured TiO_2_/TCP composite coating by plasma electrolytic oxidation. J. Alloys Compd..

[11] Mouriño V., Cattalini J.P., Boccaccini A.R. (2012). Metallic ions as therapeutic agents in tissue engineering scaffolds: An overview of their biological applications and strategies for new developments. J. R. Soc. Interface.

[12] Meretoja V.V., Tirri T., Malin M., Seppälä J.V., Närhi T.O. (2014). Enhanced osteogenicity of bioactive composites with biomimetic treatment. BioMed Res. Int..

[13] Yang L., Perez-Amodio S., Barrère-de Groot F.Y., Everts V., van Blitterswijk C.A., Habibovic P. (2010). The effects of inorganic additives to calcium phosphate on *in vitro* behavior of osteoblasts and osteoclasts. Biomaterials.

[14] Kim S., Lee J., Kim Y., Riu D.-H., Jung S., Lee Y., Chung S., Kim Y. (2003). Synthesis of Si, Mg substituted hydroxyapatites and their sintering behaviors. Biomaterials.

[15] Carlisle E.M. (1972). Silicon: An essential element for the chick. Science.

[16] Fielding G.A., Bandyopadhyay A., Bose S. (2012). Effects of silica and zinc oxide doping on mechanical and biological properties of 3D printed tricalcium phosphate tissue engineering scaffolds. Dent. Mater..

[17] Bandyopadhyay A., Petersen J., Fielding G., Banerjee S., Bose S. (2012). ZnO, SiO_2_, and SrO doping in resorbable tricalcium phosphates: Influence on strength degradation, mechanical properties, and *in vitro* bone–cell material interactions. J. Biomed. Mater. Res. Part B Appl. Biomater..

[18] Ryu H.-S., Hong K.S., Lee J.-K., Kim D.J., Lee J.H., Chang B.-S., Lee D.-H., Lee C.-K., Chung S.-S. (2004). Magnesia-doped HA/β-TCP ceramics and evaluation of their biocompatibility. Biomaterials.

[19] Hollister S.J., Maddox R.D., Taboas J.M. (2002). Optimal design and fabrication of scaffolds to mimic tissue properties and satisfy biological constraints. Biomaterials.

[20] Yeong W., Sudarmadji N., Yu H., Chua C., Leong K., Venkatraman S., Boey Y., Tan L. (2010). Porous polycaprolactone scaffold for cardiac tissue engineering fabricated by selective laser sintering. Acta Biomater..

[21] Eosoly S., Brabazon D., Lohfeld S., Looney L. (2010). Selective laser sintering of hydroxyapatite/poly-ε-caprolactone scaffolds. Acta Biomater..

[22] Miao X., Lim W.-K., Huang X., Chen Y. (2005). Preparation and characterization of interpenetrating phased TCP/HA/PLGA composites. Mater. Lett..

[23] Bose S., Tarafder S., Banerjee S.S., Davies N.M., Bandyopadhyay A. (2011). Understanding *in vivo* response and mechanical property variation in MgO, SrO and SiO_2_ doped β-TCP. Bone.

[24] Hernandez C.J., Beaupré G.S., Keller T.S., Carter D.R. (2001). The influence of bone volume fraction and ash fraction on bone strength and modulus. Bone.

[25] Impens S., Schelstraete R., Mullens S., Thijs I., Luyten J., Schrooten J. (2008). *In vitro* dissolution behavior of custom made CaP scaffolds for bone tissue engineering. Key Eng. Mater..

[26] Wei X., Ugurlu O., Ankit A., Acar H.Y., Akinc M. (2009). Dissolution behavior of Si, Zn-codoped tricalcium phosphates. Mater. Sci. Eng. C.

[27] Li X., Yasuda H., Umakoshi Y. (2006). Bioactive ceramic composites sintered from hydroxyapatite and silica at 1200 °C: Preparation, microstructures and *in vitro* bone-like layer growth. J. Mater. Sci. Mater. Med..

[28] Shuai C., Gao C., Nie Y., Hu H., Zhou Y., Peng S. (2011). Structure and properties of nano-hydroxypatite scaffolds for bone tissue engineering with a selective laser sintering system. Nanotechnology.

